# Supplementary material for: Individual and community-level determinants of knowledge of ovulatory cycle among women of childbearing age in Ethiopia: A multilevel analysis based on 2016 Ethiopian Demographic and Health Survey
Source: PLoS One. 2021 Sep 2;16(9):e0254094. doi: 10.1371/journal.pone.0254094 (PMC8412270; doi:10.1371/journal.pone.0254094)
Supplement: Attachment — Submitted filename: Response to Reviewers.docx [file pone.0254094.s002.docx]

**Point-by-point response to the editor and reviewers**

**Academic editor’s comment:** I have to note with much regret that this submission carries out almost identical analysis with the same data as another paper that had been published before the initial submission. Note that PLOS ONE does not accept for publication studies that have already been published elsewhere in the peer-reviewed literature and if a submitted study replicates or is very similar to previous work, authors must provide a sound scientific rationale for the submitted work and clearly reference and discuss the existing literature. Submissions that replicate or are derivative of existing work will likely be rejected if authors do not provide adequate justification. I know this should have been identified in the previous round and I apologize for the late feedback in that respect. Ref: <https://doi.org/10.2147/IJWH.S267675> . You must discuss this article among the literature review and incorporate its findings. If you can make a convincing case of why the article should be considered despite this, the article would be evaluated further.

**Authors’ reply:** The previous study used the Ethiopian Demographic and Health Survey (EDHS) 2016 [20]. The previous study did not consider the community level factors (they consider factors only at the individual-level; even they did not use multilevel analysis which is appropriate for hierarchical data), there was no weighting to account for appropriate estimation, and there were missing of essential variables such as media exposure which plays a crucial role for the provision of information including reproductive health. It is useful to consider the community-level factors for designing interventional strategies not only based from the individual-level factors. In a summary, our study differs from the previous one by considering the following;

1. Our study used sampling weight (using women's sampling weight) to account for the non-proportional allocation of the sample in each region and the potential variations in the response rates, as well as to get an appropriate statistical estimate. Besides, stratification was used to account for the survey design
2. We included community-level factors (community-education, community poverty and community media exposure). As explained above, strategies should based from community level factors besides individual level factors.
3. We used multilevel multivariable analysis; clustering effect was performed due to the hierarchical nature of the DHS data. Intra-class correlation coefficient (ICC) and median odds ratio (MOR) were computed to choose appropriate model. We fitted four models; null model/ (containing only the outcome variable), the model I (with individual-level factors only), model II (with community-level factors only), and model III (with the individual- and community-level factors simultaneously). Finally, we used the third model by looking the lowest deviance.
4. In addition to the individual factors included by the previous study, we included media exposure which is crucial factor for the provision of information including reproductive health knowledge.

For more information, we included it in the revised manuscript Line#70-6 and in the discussion section

**Reviewers’ comments to the Authors
Authors’ reply to the reviewer#1:** We acknowledge the reviewer for accepting our previous replies to the comments. In this phase, there is no comment from the reviewer#1 to be addressed.

**Authors’ reply to the reviewer#2**

**Reviewer #2 (comments):** The authors have effectively addressed the majority of the comments provided by reviewers. However, a few comments were only responded to by the authors without effecting the needed revisions in the manuscript.

1. Authors stated that data used for the study were included in the manuscript but were not actually included. Authors should provide the link to the dhsprogram data for EDHS 2016 in the editorial manager system in place of the "All relevant data are included in the manuscript" statement.

**Authors’ reply:** We provided the link to DHS program in the editorial system instead of “"All relevant data are included in the manuscript" statement. All the data were included in the manuscript. There is no restriction. The data can be accessed from the DHS website after reasonable request (<https://dhsprogram.com/Data/terms-of-use.cfm>)

2. The existing literature supporting the variables was only explained but not provided as recommended. Some of these citations should be provided in the Study Variables section to support the variable selection.

**Authors’ reply:** Thank you very much. We included few citations to the variables section to indicate where we get the variables to consider them as independent (Line#139).

3. The first paragraph of the "Study variables" has a different spacing from the rest of the manuscript.

**Authors’ reply:** Thank you. We corrected as suggested (formerly it was 1.5 and now we make it double spacing).

4. The "BMC Series" format of manuscript presented after the conclusion section should be removed (List of abbreviations to author contributions, EXCEPT Acknowledgements) as all these are pre-filled in and generated from the editorial manager system onto the final paper.

**Authors’ reply:** Thank you. We removed as advised.

5. Some clear grammatical problems remain in the manuscript and authors have to submit the manuscript for expert copy-editing before re-submission.

**Authors’ reply:** We invited an expert person from the department of literature at the University of Gondar for the revision of the grammatical and standard use of English language. Then, we incorporated the points from the invited person and all the authors revised the manuscript for the correction of grammatical flaws. We hope, we have made improvements in the revised manuscript.
